# Antimicrobial Natural Products

**DOI:** 10.3390/antibiotics11121765

**Published:** 2022-12-07

**Authors:** Fuhang Song

**Affiliations:** School of Light Industry, Beijing Technology and Business University, Beijing 100048, China; songfuhang@btbu.edu.cn

Infectious diseases, resulting from microbial pathogens, are one of the major causes of morbidity and mortality worldwide. Natural products derived from foods, microbes, and plants played an important role in combating infections before the discovery of antibiotics. The start of the antibiotics era was marked by the clinical introduction of penicillin, which was the first antibiotic, discovered in 1928 by Alexander Fleming in the green mold *Penicillium notatum*. During the golden age of natural-product drug discovery (from the 1950s to the 1960s), the Nobel Prize in Physiology or Medicine was awarded to Alexander Fleming, Ernst B. Chain, and Sir Howard Florey in 1945 for their discovery of penicillin, and to Selman A. Waksman in 1952 for the discovery of streptomycin. The 2015 Nobel Prize in Physiology or Medicine was awarded to William C. Campbell and Satoshi Omura for their discovery of avermectins, a natural microbial product, and to Youyou Tu for her discovery of the natural plant product artemisinin; this heralded a new golden age of natural-product drug discovery [1].

This Special Issue features papers by experts working in natural product research, focusing on the control of microbial pathogens using natural products.

Propolis is a traditional, potentially medicinal product with several health benefits. Marta Peixoto and co-workers investigated the antimicrobial potential of ethanol extracts of propolis collected in Pereiro over a 5-year period (2011–2015) and two distinct apiaries/regions—Pereiro and Gerês—in selected years [2]. The results showed that a mixture of propolis and ethanol extracts had particularly interesting effects on *Bacillus subtilis*, *Propionibacterium acnes*, and *Staphylococcus aureus*. For *Saccharomyces cerevisiae* fungus, the mixtures mostly displayed MIC values similar to those of the most active single extract, except for mP (P11.EE + P13.EE), which was more active than the single extracts. This paper was a first attempt to evaluate the chemical profiles and bioactivity of mixed propolis samples from different years and regions. The findings of the study suggest great medicinal potential for propolis and are an important contribution to its valorization and standardization.

Bryophytes are important resources with the potential to produce unique natural compounds with antimicrobial properties. Valeeva and co-workers evaluated and characterized the antibacterial activity of intracellular and extracellular metabolites produced by the model mosses *Physcomitrium patens* and *Sphagnum fallax* [3]. Both polar (methanol-based) and non-polar (hexane-based) extracts of the two mosses inhibited the growth of Gram-negative *P. syringae* DC3000 bacteria. However, the bioactive metabolites were not stable during the extraction process; moreover, bioactivity completely ceased after lyophilization, while the secreted exudate fractions remained stable throughout the experiments. Exudates from both *P. patens* strains displayed selective high antimicrobial activity against Gram-positive *S. aureus*, *Enterococcus faecium*, and *Streptococcus pyogenes*. No antibacterial activity was observed with any exudates against Gram-negative bacteria *Salmonella*, *S. marcescens*, or *Escherichia coli*. 

Natural products from fungi play a prominent role in the development of new drugs. Han and co-workers reported that the full molecular network of crude extracts of *Aspergillus westerdijkiae* L1295 showed several independent families of molecules; moreover, detailed analysis of the molecular network revealed a cluster with 19 nodes representing a peptide family, showing MS/MS patterns containing the dipeptide [Ala-Phe] fragment. Guided by MS/MS and molecular networking, two new cyclic tetrapeptides (CTPs) (westertides A and B) with eight known compounds (ochratoxin A, ochratoxin A methyl ester, circumdatin F, circumdatin G, stachyline B, westerdijkin A, mellein, and 3-hydroxymellein) were identified from the fungus *A. westerdijkiae*, guided by OSMAC (one strain–many compounds) strategies [4]. Westertides A and B showed strong synergistic antifungal activity against *Candida albicans* with rapamycin. Furthermore, westertide A showed weak (histone deacetylase, HDAC) inhibitory activity.

Halogen substituents significantly impact the bioactivity and reactivity of organic compounds, so halogenated compounds play a profound role in the pharmaceutical industry. In the investigations by Luo and co-worders, *GedL*, a free-standing phenol flavin-dependent halogenase (FDH) from *A. terreus* NIH2624, is involved in the biosynthesis of geodin, and halogenates the substrate in late-stage biosynthesis. A flavin-dependent halogenase gene, *ptaK*, was identified from a cryptic BGC of endolichenic *Pestalotiopsis rhododendri* LF-19-12 via genome mining. A group of potential halogenated compounds with characteristic isotope patterns of two chloride atoms were detected in the crude extract of *P. rhododendri* LF-19-12 cultured in M2 medium using LC-MS and OSMAC strategies [5]. Then, two pairs of atropisomers (pestalachlorides A1a/A1b and A2a/A2b), together with two known compounds (pestalachloride A and SB87-H), were identified from *P. rhododendri* LF-19-12. Pestalachlorides exhibited antibacterial activity against drug-sensitive and drug-resistant *S. aureus* and *E. faecium*, with MIC values ranging from 4 mg/mL to 32 mg/mL.

Fungi from marine-derived environments are promising resources for the discovery of new chemical entries. Song and co-workers investigated the chemical constituents of the marine-derived *Talaromyces* sp. Fungus, isolated from a mud sample collected from the intertidal zones of the Yellow Sea in Qingdao, China. Three new compounds, including two new polyketide-derived oligophenalenone dimers (bacillisporins K and L) and one xanthoradone dimer (rugulosin D), along with four known compounds (bacillisporin B, macrosporusone D, rugulosin A, and penicillide), characterized this marine-derived fungus [6]. Bacillisporins K and L, bacillisporin B, macrosporusone D, rugulosin A, and penicillide exhibited antibacterial activity against *S. aureus*, with MIC values of 12.5, 25, 12.5, 6.25, 0.195, and 100 μg/mL, respectively. 

Microorganism interactions offer each of the strains specific metabolic potential. Wang and co-workers studied the effect of co-culture on the secondary metabolites and bioactivity of two marine strains, *A. terreus* C23-3 and *A. unguis* DLEP2008001 [7]. Both of the strains grew well and produced metabolites when inoculated simultaneously, and *A. terreus* seemed to be more strongly induced by live *A. unguis*. Under some conditions, the extracts of co-culture showed higher antimicrobial activity than the axenic cultures. Different yields in the co-cultures vs. the corresponding axenic culture of fifteen MS-detectable and/or UV-active peaks were detected via LC-PDA-MS/MS analysis. Both strains produced chemical ‘weapons’ for antagonism. This study revealed the different responses of two *Aspergillus* strains in co-culture, which highlights new opportunities for antibiotic discovery.

Kartsev and co-workers synthesized a series of heteroaryl(aryl) thiazole derivatives based on a molecular hybridization approach [8]. Three of the synthesized compounds displayed antibacterial activity against pathogenic strains, including methicillin-resistant *S. aureus*, *P. aeruginosa*, and *E. coli*, with higher potential than the reference drug ampicillin. Some of the compounds exhibited better antifungal activity; the best compound was 4-butyl-1-hydroxy-*N*-(6-methylbenzo[d]thiazol-2-yl)-3-oxo-3,4-dihydronaphthalene-2-carboxamide with an MIC of 0.06–0.23 mg/mL and an MFC of 0.11–0.47 mg/mL. Docking studies revealed that the putative mechanisms are inhibition of the *E. coli* MurB enzyme and the inhibition of fungal 14a-lanosterol demethylase.

Drug delivery offers improvements to the therapeutic effects and systemic side effects of administered drugs. Khattak and co-workers studied the synergistic antibacterial activity of a chitosan-coated bacitracin cream under different in vitro characteristics such as rheology, pH, viscosity, and drug content. They revealed that the zones of inhibition in simple bacitracin-loaded cream were significantly smaller than those in chitosan-decorated bacitracin-loaded cream, indicating that chitosan synergistically improves the antimicrobial activity of bacitracin [9]. This contribution provided an effective method for the topical management of skin infections and wound healing.

Tran and co-workers focused on the antimicrobial compounds produced by *Bacillus strains*, their proposed mechanisms of action, and any research gaps in the mechanisms of these compounds. Omics approaches were also reviewed to clarify the mechanisms behind Bacillus probiotics [10].

Li and co-workers focused on the biocontrol of *C. albicans* by antagonistic microorganisms and bioactive compounds. In this review, the authors reported the bacteriostatic behavior of different antagonistic microorganisms (bacteria and fungi) against *C. albicans*. Moreover, they reviewed the natural products produced by microorganisms with unique structures and antifungal activity and their possible inhibitory mechanisms [11]. 

Maja Urošević and co-workers reviewed recent research on the biological and pharmaceutical aspects of curcumin, methods of sample preparation for its isolation, analytical methods for its identification and quantification in different matrices, and different techniques for developing formulations [12].

The emergence of multi-drug-resistant microbes is one of the most critical medical problems, and has prompted valuable contributions to new antibacterial drug development. This Special Issue presents multidisciplinary research focused on natural products with the aim of strengthening antimicrobial studies. The contributions collected herein provide valuable knowledge for researchers working in the field of natural-product chemistry.
